# Suramin Alleviates Glomerular Injury and Inflammation in the Remnant Kidney

**DOI:** 10.1371/journal.pone.0036194

**Published:** 2012-04-27

**Authors:** Na Liu, Song He, Evelyn Tolbert, Rujun Gong, George Bayliss, Shougang Zhuang

**Affiliations:** 1 Department of Nephrology, Shanghai East Hospital, Tongji University School of Medicine, Shanghai, China; 2 Department of Medicine, Rhode Island Hospital, Alpert Medical School, Brown University, Providence, Rhode Island, United States of America; 3 Department of Laboratory Medicine, Shekou People's Hospital, Shenzhen, Guangdong Providence, China; Centro de Pesquisa Rene Rachou/Fundação Oswaldo Cruz (Fiocruz-Minas), Brazil

## Abstract

**Background:**

Recently, we demonstrated that suramin, a compound that inhibits the interaction of multiple cytokines/growth factors with their receptors, inhibits activation and proliferation of renal interstitial fibroblasts, and attenuates the development of renal interstitial fibrosis in the murine model of unilateral ureteral obstruction (UUO). However, it remains unclear whether suramin can alleviate glomerular and vascular lesions, which are not typical pathological changes in the UUO model. So we tested the efficacy of suramin in the remnant kidney after 5/6 nephrectomy, a model characterized by the slow development of glomerulosclerosis, vascular sclerosis, tubulointerstitial fibrosis and renal inflammation, mimicking human disease.

**Methods/Findings:**

5/6 of normal renal mass was surgically ablated in male rats. On the second week after surgery, rats were randomly divided into suramin treatment and non-treatment groups. Suramin was given at 10 mg/kg once per week for two weeks. In the remnant kidney of mice receiving suramin, glomerulosclerosis and vascular sclerosis as well as inflammation were ameliorated. Suramin also attenuated tubular expression of two chemokines, monocyte chemoattractant protein-1 and regulated upon expression normal T cell expressed and secreted (RANTES). After renal mass ablation, several intracellular molecules associated with renal fibrosis, including NF-kappaB p65, Smad-3, signal transducer and activator of transcription-3 and extracellular regulated kinase 1/2, are phosphorylated; suramin treatment inhibited their phosphorylation. Futhermore, suramin abolished renal ablation-induced phosphorylation of epidermal growth factor receptor and platelet derived growth factor receptor, two receptors that mediate renal fibrosis.

**Conclusions and Significance:**

These findings suggest that suramin attenuates glomerular and vascular injury and reduces inflammatory responses by suppression of multiple growth factor receptor-mediated profibrotic signaling pathways. Therefore, suramin may be a useful drug in preventing the fibrosis and sclerosis that characterizes progression of chronic kidney disease.

## Introduction

The pathogenesis of chronic kidney disease (CKD) in most cases involves a complex interaction of hemodynamic and inflammatory processes that leads to glomerular and vascular sclerosis and tubulointerstitial scarring with subsequent progression toward ESRD. Although therapeutic interventions such as the blockade of the rennin-angiotensin-aldosterone system and immunosuppressive drugs slow the progression of renal disease and promote renoprotective effects in experimental animal models [1], [2] and in humans [3], [4], these strategies cannot halt the progression of renal fibrosis and scarring. The development and progression of renal fibrosis is primarily involved in the differentiation of renal fibroblasts into myofibroblasts and infiltration of inflammatory cells, regulated by numerous cytokines and growth factors. Thus a therapeutic intervention that blocks the activation of multiple cytokine and growth factor receptors might improve antifibrotic effects and help slow progression of chronic nephropathies.

Suramin, a FDA-approved drug that inhibits the interaction of multiple cytokines and growth factors with their receptors [5], [6], [7], [8], [9], may represent a novel therapeutic option for blocking fibrogenesis. This drug was Initially developed as a treatment for trypanosomiaisis and selected malignancies and metastatic diseases [5], [6], [7], [8], [9]. Recently, several studies have shown that administration of suramin protects against the development of muscle and liver fibrosis in animal models [10], [11]. By using a mouse model of unilateral urethral obstruction (UUO), we also demonstrated that suramin is effective in attenuating renal interstitial fibrosis [12], [13]. This protective effect of suramin was further confirmed in a rat model of remnant kidney disease as indicated by preservation of renal tissue architecture, prevention of progressive renal interstitial fibrosis and improvement of renal function [12]. However, it remains unclear whether suramin is able to attenuate glomerular and vascular sclerosis as well as inflammation, in particular, macrophage infiltration in the remnant kidney.

The remnant kidney after 5/6 nephrectomy is a classic model that mostly mimics CKD in humans [14], [15]. Histological studies of remnant kidney tissue have indicated a complex response consisting primarily of three steps: a hypertrophic phase, a quiescent phase with minimal histological alterations, and finally the development of segmental glomerular sclerosis and tubulointerstitial fibrosis [16], [17]. Hyperfiltration has been identified as a significant contributor to the hypertrophy and glomerulosclerosis and macrophages play a key role in renal injury induced by glomerular hyperfiltration following reduction of kidney mass by ablation [18]. Macrophages localize to glomeruli and the interstitium via interactions with several chemokines, including monocyte chemo attractant protein-1(MCP-1), and regulated upon activation, normal upon expression normal T cell expressed and secreted (RANTES) [18], [19], [20]. This proinflammatory microenvironment promotes renal scarring [14], [15]. In the kidney, tubular epithelial cells are considered to be a prominent source of chemokines [21]. MCP-1 and RANTES are two key chemokines that recruit monocytes/macrophages to the kidney [22], [23].

Many mediators, including transformation growth factor-beta1 (TGF-beta1), epidermal growth factor (EGF), and platelet-derived growth factor (PDGF), are also upregulated in glomeruli and tubules and are believed to be involved in the progression of glomerular sclerosis and tubulointerstitial fibrosis [24], [25], [26]. The interaction of cytokines/growth factors with their receptors initiates different signaling pathways, leading to the activation of multiple transcriptional factors such as NF-kappaB and signal transducer and activator of transcription 3 (STAT3) that regulate various genes related to the activation of renal fibroblasts and initiation of inflammation [27]. In addition, activation of smad3, a key intracellular mediator in TGF-beta signaling, is induced in different models of renal fibrosis [12]. Recently, we have shown that suramin treatment can block phosphorylation of EGF receptor (EGFR), PDGF receptor (PDGFR), and smad3, However, it is unknown whether suramin is able to interfere with those signaling pathways and inactivation of NF-kappaB in the remnant kidney.

In this study, we examined the effect of suramin on glomerular and vascular sclerosis, and inflammatory cell infiltration in the remnant kidney model. Further, we investigated the effect of suramin on the expression of MCP-1 and RANTES and the activation of growth factor receptors/intracellular signaling pathways associated with the development of progressive renal injury.

## Results

### Suramin inhibits glomerular and vascular sclerosis in rat remnant kidneys

**Figure 1:**
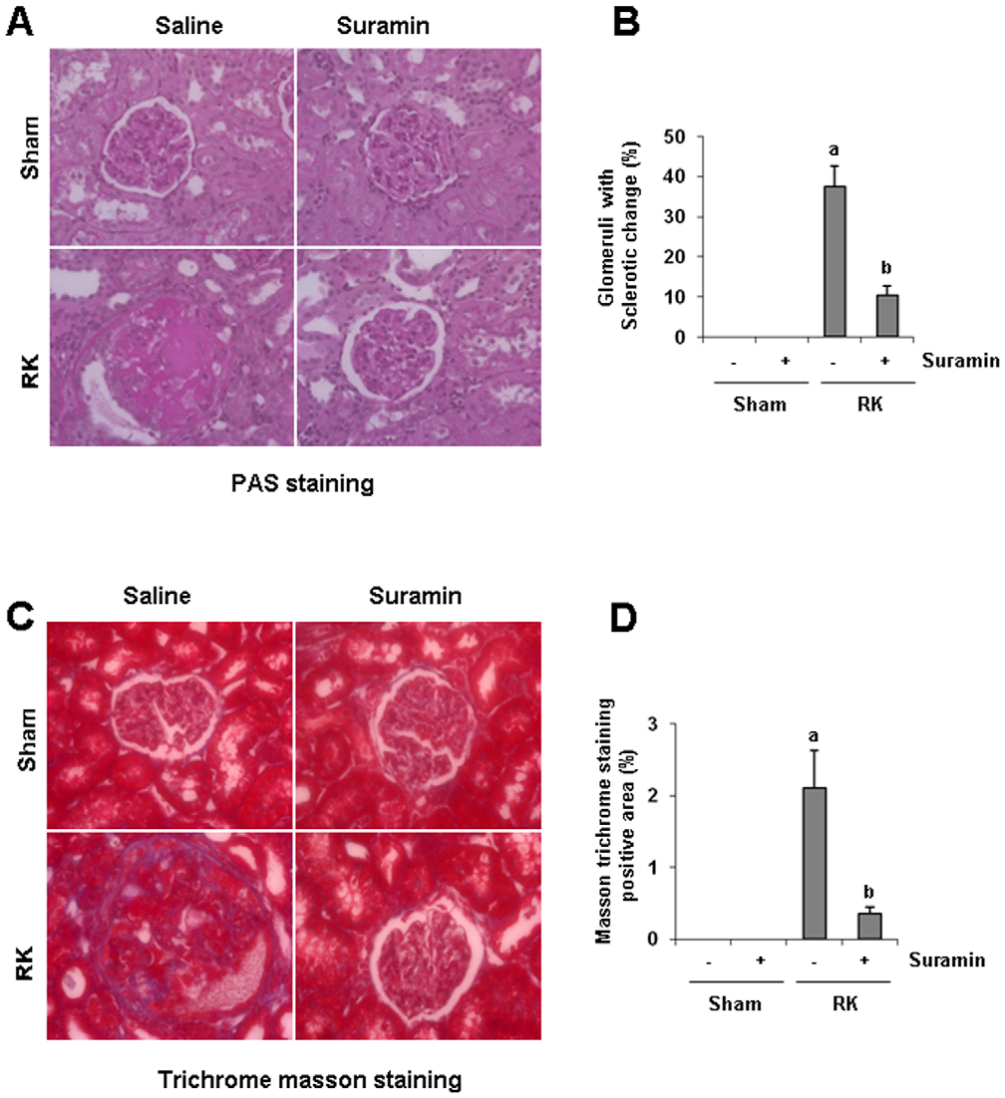
Effect of suramin on glomerular sclerosis in rat remnant kidney. (A) Photomicrographs Photomicrographys (400×) illustrating periodic acid-Schiff-stained sections of kidney tissue on week 4 after various treatments as indicated. (B) Photomicrographs (400×) illustrating trichrome stained kidney tissue on week 4 after various treatments. RK, remnant kidney.

**Figure 2:**
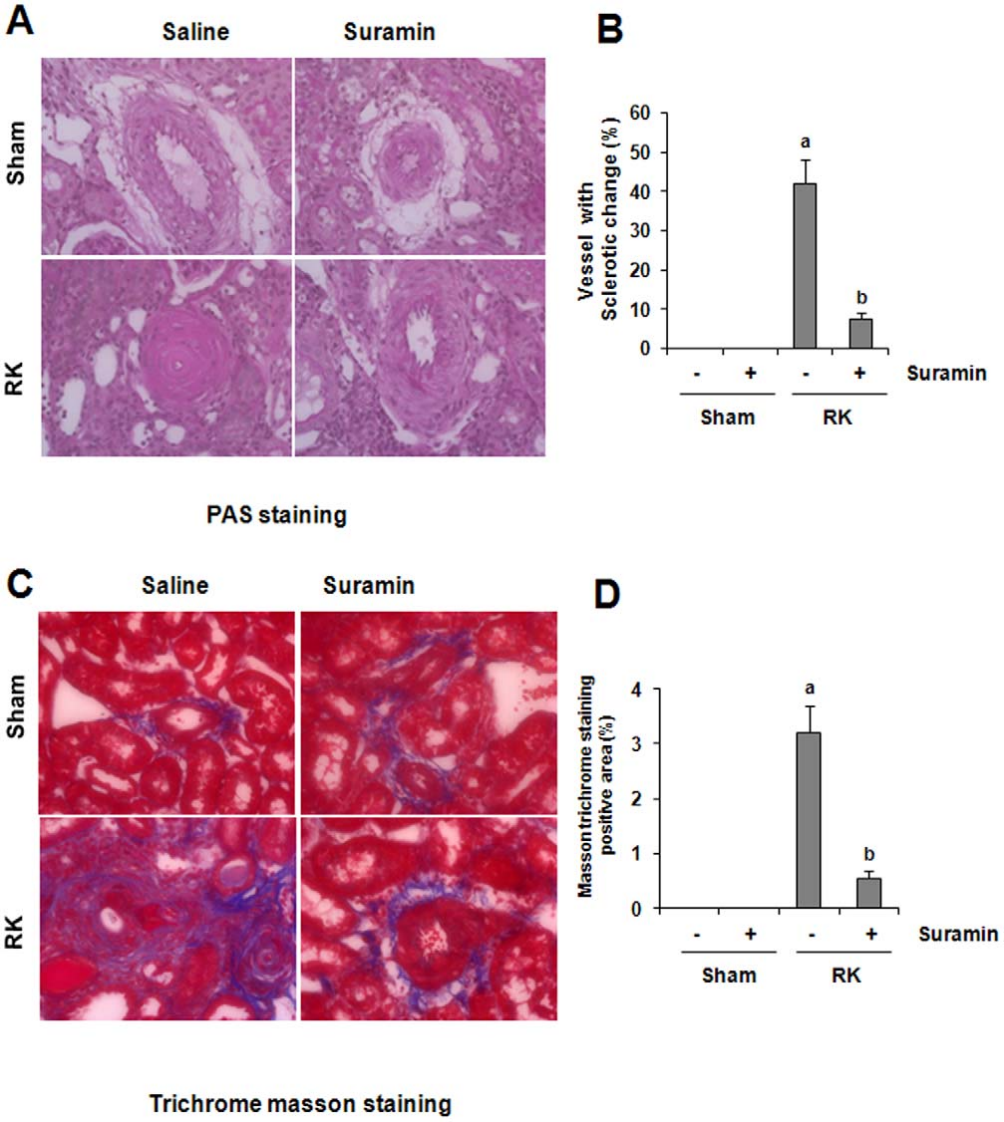
Effect of suramin on vascular sclerosis in rat remnant kidney. (A) Photomicrographs (400×) illustrating periodic acid-Schiff-stained sections of kidney tissue on week 4 after various treatments as indicated. (B) Photomicrographs (400×) illustrating trichrome stained kidney tissue on week 4 after various treatments. RK, remnant kidney.

We recently showed that suramin prevented progressive renal injury as demonstrated by inhibition of a rise in 24 hour-protein excretion and a rise in serum creatinine by preserving renal tissue architecture and preventing the development of renal interstitial fibrosis in the rat model of remnant kidney disease [12]. As glomerular and vascular sclerosis are also two major pathogenetic changes in the model of remnant kidneys, we further examined the effect of suramin on those events. As shown in Figure 1 and 2, after 4-weeks of renal ablation, 15% of glomeruli were damaged and displayed segmental and focal solidification and sclerosis of the glomerular tuft (Fig. 1A). Forty percent of renal arteries also show an onion skin-like change, indicating vascular sclerosis in the remnant kidney (Fig. 2A). By comparison, very few sclerotic glomeruli and arteries were observed in the kidney of animals that received suramin treatment. There were no obvious pathological changes in the kidney of both sham and suramin alone-treated animals.

Masson trichrome staining showed increased deposition of collagen fibrils in glomeruli and blood vessels following the 5/6 renal ablation (Fig. 1B, 2B). Administration of suramin diminished deposition of collagen fibrils in glomeruli and blood vessels. Perivascular collagen fibril deposition around small arteries also increased, but was suppressed by suramin treatment. Notably, in the normal kidney, expression of ECM protein was seen in perivascular areas (positive on Masson trichome staining) and was not affected by suramin (Fig. 2B). This data, together with our previous observation that suramin inhibits the development of renal interstitial fibrosis, suggests that suramin application has renoprotective properties and is able to prevent the development of fibrotic lesions in all areas including glomeruli and arteries in the remnant kidney.

### Suramin reduces infiltration of monocytes/macrophages to the remnant kidney

**Figure 3:**
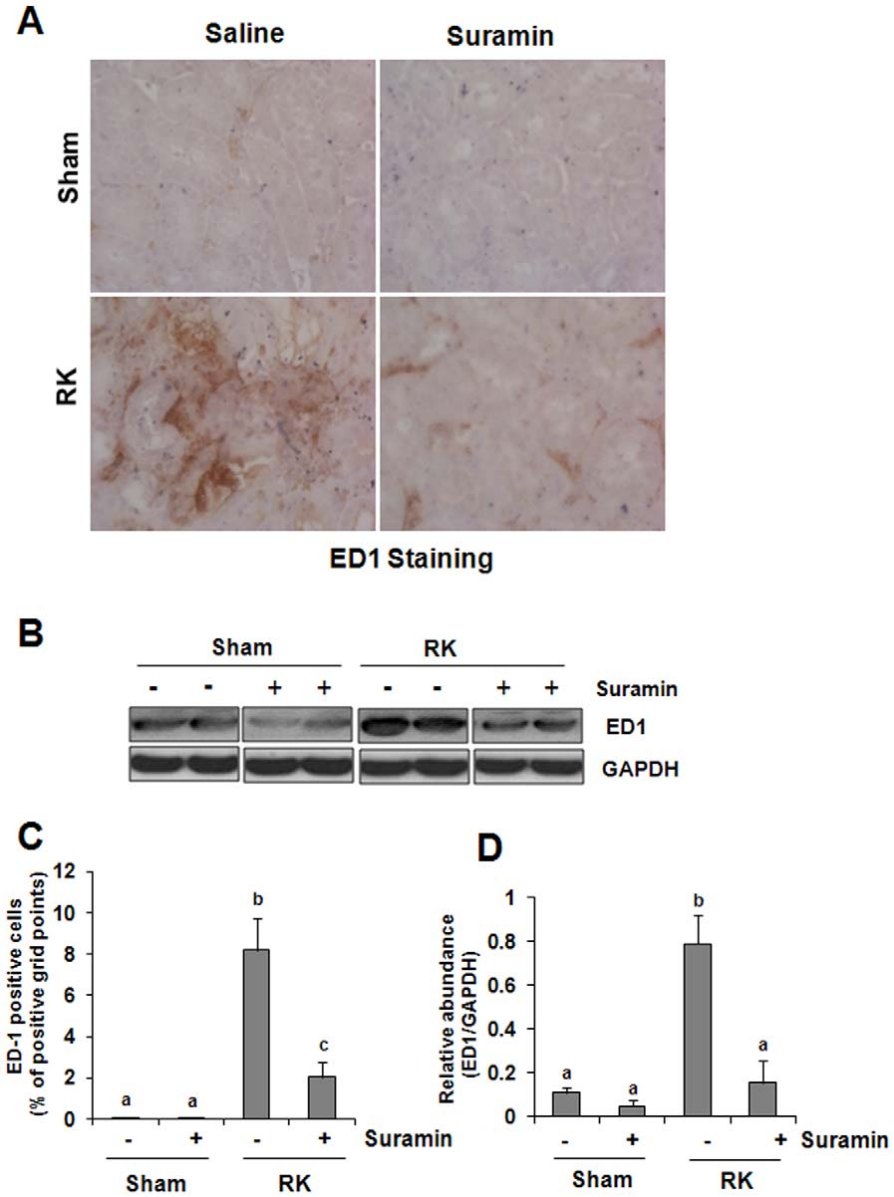
Effect of suramin on monocytes/microphage accumulation in rat remnant kidney. (A) Photomicrographs (400×) illustrating ED1-stained sections of kidney tissue on week 4 after various treatments as indicated. (B) Kidney tissue lysates were subjected to immunoblot analysis with specific antibodies against ED1 and GAPDH. (C) ED1 staining graphic presentation of quantitative data. (D) Expression levels of ED1 was quantified by densitometry and normalized with GAPDH. Data are represented as the mean ± S.E.M (n = 6). Means with different superscript letters are significantly different from one another (*P*<0.05).

Kidney fibrosis is almost always preceded by and closely associated with chronic interstitial inflammation [23], [28], [29], [30]. Macrophage infiltration and proliferation functionally contributes to the fibrotic process after injury [31], [32]. To determine the effect of suramin on these processes, we first conducted ED-1 staining, which detects active monocytes/macrophages. Figure 3A showed that monocyte/macrophage infiltration was increased in the remnant kidney compared with the sham-operated kidney (Fig. 3A). Suramin treatment significantly reduced the number of monocytes/macrophages (Fig. 3A). Next, we determined the expression levels of ED-1 in the kidney tissue by immunoblot analysis. ED1 was detected in the normal kidney, and its levels were increased in the remnant kidney. Suramin treatment also reduced ED1 expression to the basal level in the tissue of the remnant kidney. This data, together with our recent observation that neutrophil numbers are reduced in the obstructed kidney treated with suramin [12], suggest that suramin is also able to suppress infiltration of inflammatory cells into the damaged kidney in chronic injury.

### Suramin attenuates the expression of MCP-1 and RANTES after renal ablation

**Figure 4:**
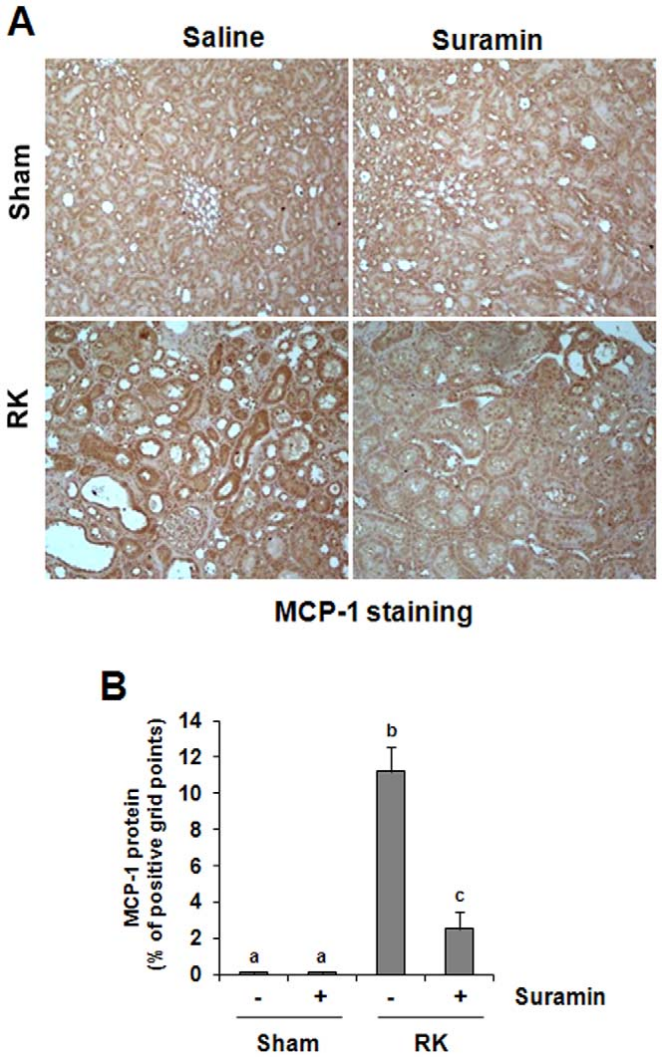
Effect of suramin on MCP-1 expression in rat remnant kidney. (A) Photomicrographs (400×) illustrating MCP-1-stained sections of kidney tissue on week 4 after various treatments as indicated. (B) MCP-1 staining graphic presentation of quantitative data. Data are represented as the mean ± S.E.M (n = 6). Means with different superscript letters are significantly different from one another (*P*<0.05).

**Figure 5:**
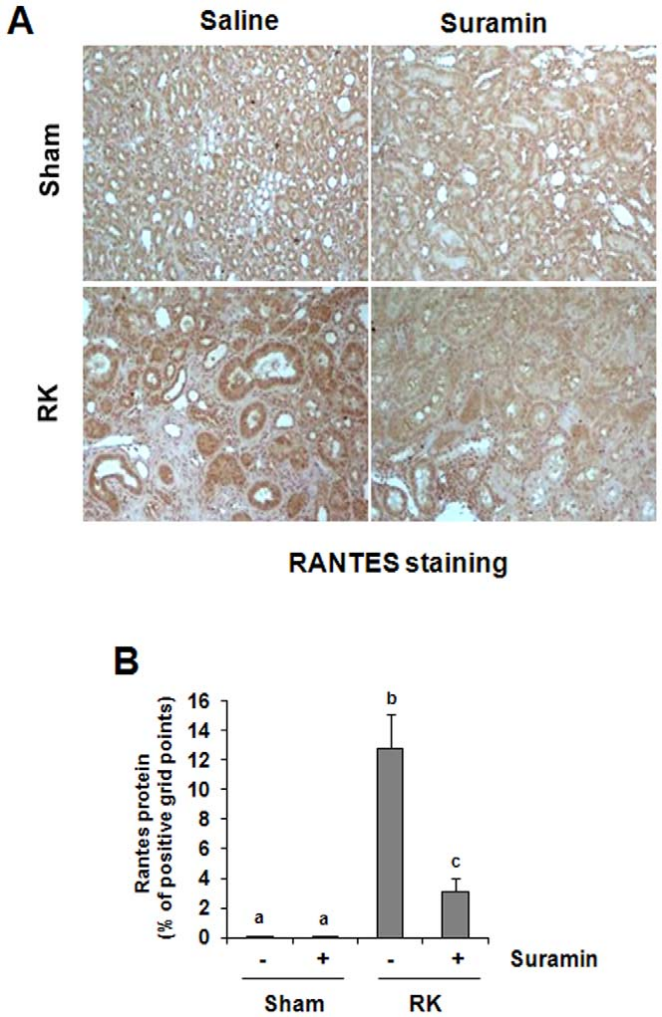
Effect of suramin on RANTES expression in rat remnant kidney. (A) Photomicrographs (400×) illustrating RANTES-stained sections of kidney tissue on week 4 after various treatments as indicated. (B) RANTES staining graphic presentation of quantitative data. Data are represented as the mean ± S.E.M (n = 6). Means with different superscript letters are significantly different from one another (*P*<0.05).

MCP-1 and RANTES are two chemokines that play an important role in attracting inflammatory cells including monocytes/macrophages to sites of inflammation, and contributes to renal interstitial fibrosis [33], [34]. In the kidney, tubular epithelial cells (TEC) are considered a prominent source of chemokines [21]. Thus, we examined their expression by immuohistochemistry. A low level of MCP-1 was detected in the sham-operated with or without treatment with suramin (Fig. 4A). However, abundant expression of MCP-1 was detected in the remnant kidney, primarily localized in tubular cells (Fig. 4B). Administration of suramin significantly decreased MCP-1 expression levels (Fig. 4A,B). A basal level of RANTES protein was observed in control kidneys (sham alone or sham plus suramin treatment); and its expression was elevated in the remnant kidney. Like MCP-1, RANTES was also predominantly expressed in the renal tubules (Fig. 5A). Suramin treatment reduced its expression (Fig. 5B). These results, in conjunction with our recent observations that suramin inhibits expression of multiple cytokines including TGF-beta1, TNF-alpha, Interleukin-1, ICAM-1 [12] in the model of UUO, suggest that suramin has a strong inhibitory effect on inflammatory responses in the renal kidney.

### Suramin reduces NF-kappaB activation in the rat remnant kidney

**Figure 6:**
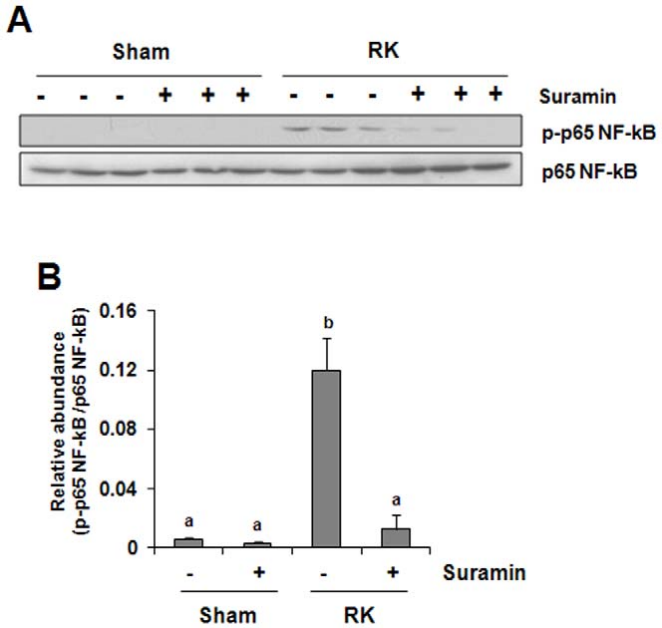
Effect of suramin on NF-kappaB activation in rat remnant kidney. Kidney tissue lysates were subjected to immunoblot analysis with specific antibodies against phospho-p65 NF-kappaB, p65NF-kappaB. Expression levels of p-p65 NF-kappaB(B) were quantified by densitometry and normalized with p65 NF-kappaB. Data are represented as the mean ± S.E.M (n = 6). Means with different superscript letters are significantly different from one another (*P*<0.05).

NF-kappaB is a ubiquitous eukaryotic transcription factor that regulates gene expression of cytokines and enzymes involved in controlling inflammatory responses [35]. Phosphorylation of the p65 unit of NF-kappaB initiates its activation and plays an important role in regulating the specificity of the NF-kappaB-dependent gene expression [36]. Suramin may regulate expression of multiple cytokines and chemokines, through activation of NF-kappaB. To test this hypothesis, we determined the phosphorylation of p65 NF-kappaB in rat remnant kidney treated with/without suramin by Western blot analysis. Figure 6 shows that the phosphorylated NF-kappaB p65 was not detectable in the kidney of sham-operated rats, but significantly increased in the remnant kidney. Suramin treatment decreased its phosphorylation. Expression of total p65 NF-kappaB was not affected by renal ablation and suramin. These data suggest that suramin is effective in inhibiting NF-kappaB activation in the remnant kidney, providing the molecular basis for decreased expression of chemokines/cytokines in the injured kidney treated with suramin after urethral obstruction and 5/6 renal ablation.

### Suramin abolishes phosphorylation of STAT3 and ERK1/2 renal ablation

**Figure 7:**
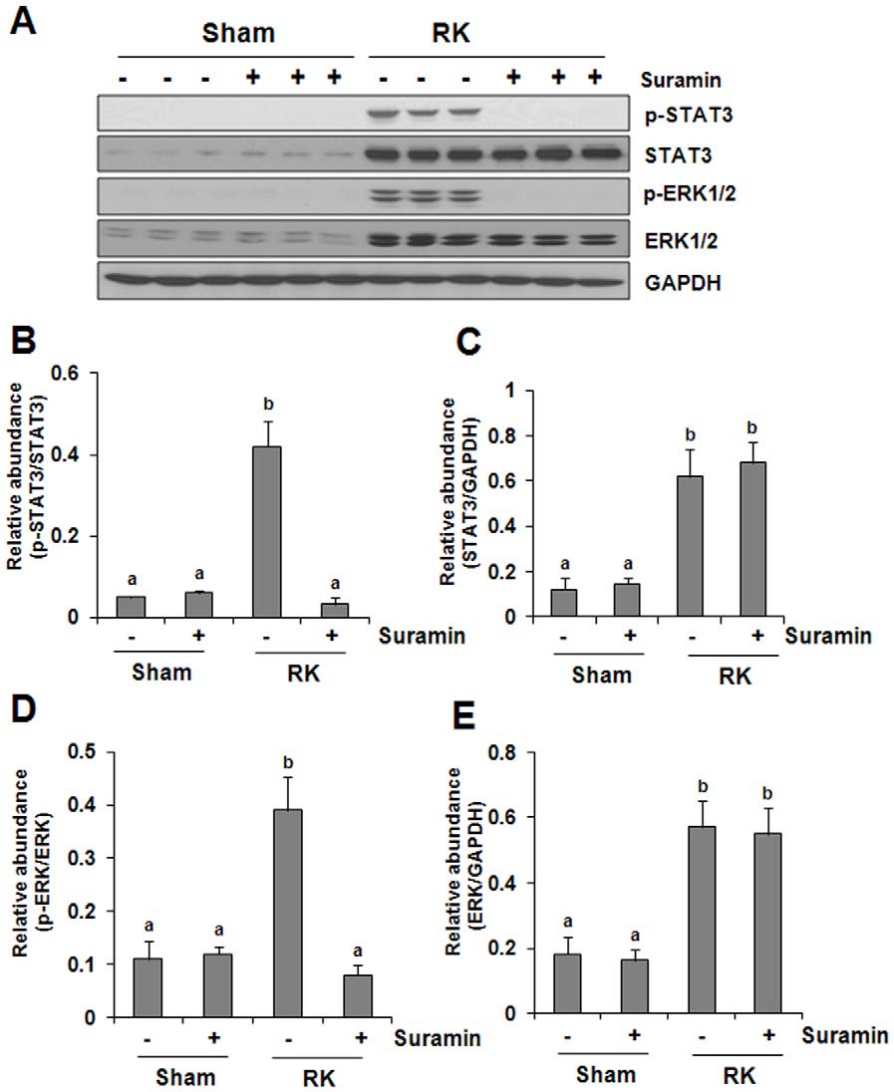
Effect of suramin on phosphorylation of STAT3 and ERK1/2 in rat remnant kidney. (A) Kidney tissue lysates were subjected to immunoblot analysis with specific antibodies against phospho-STAT3 (p-STAT3), STAT3, phospho-ERK1/2 (p-ERK1/2), ERK1/2. Expression levels of p-STAT3 (B), STAT3 (C), p-ERK1/2 (D), ERK1/2 (E) were quantified by densitometry and normalized with GAPDH. Data are represented as the mean ± S.E.M (n = 6). Means with different superscript letters are significantly different from one another (*P*<0.05).

In addition to NF-kappaB, STAT3 and ERK1/2 pathways also play an important role in modulating inflammatory responses [37], [38] and renal fibroblast proliferation. We thus further examined the effect of suramin on STAT3 and ERK1/2 phosphorylation in the remnant kidney. As shown in Fig. 7, increased STAT3 and ERK1/2 phosphorylation (activation) was detected in rat remnant kidney and suramin treatment abolished this response. Remnant kidney injury also increased expression levels of total STAT3 and ERK1/2, but their expression was not affected by suramin. This data indicates that suramin may also suppress renal inflammation and fibroblast activation by interfering with these two signaling pathways.

### Suramin abrogates phosphorylation of EGFR and PDGFR in rat remnant kidney

**Figure 8:**
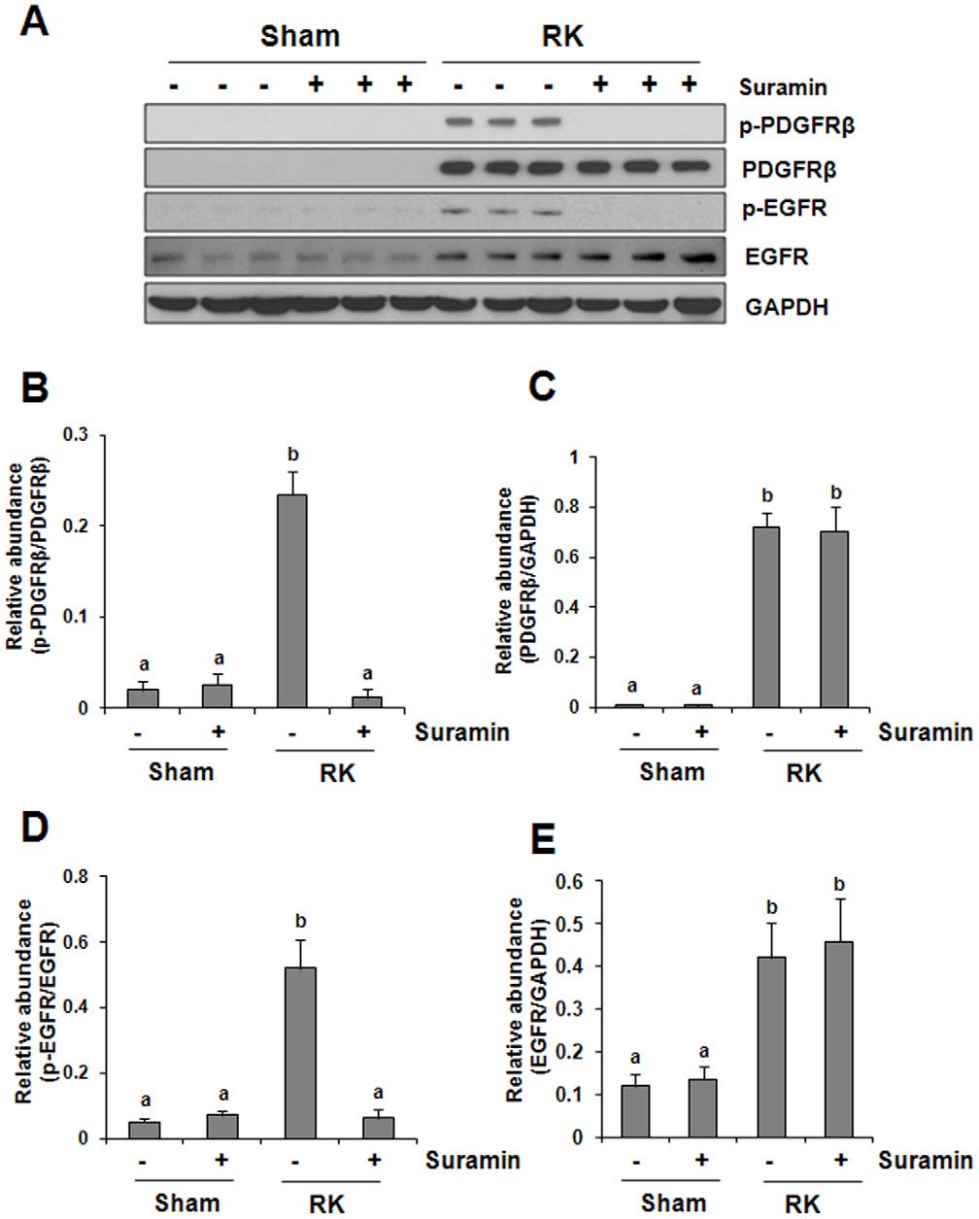
Effect of suramin on phosphorylation of PDGF receptor and EGF receptor in rat remnant kidney. (A) Kidney tissue lysates were subjected to immunoblot analysis with specific antibodies against phospho-PDGF receptor-beta (p-PDGFR-beta), PDGF receptor, phospho-EGF receptor (p-EGFR), EGF receptor (EGFR). Expression levels of p-PDGFR-beta(B), PDGFR-beta (C), p-EGFR (D), EGFR (E) were quantified by densitometry and normalized with GAPDH. Data are represented as the mean ± S.E.M (n = 6). Means with different superscript letters are significantly different from one another (*P*<0.05).

Activation of intracellular signaling pathways is usually secondary to the activation of membrane receptors. EGFR and PDGFR have been shown to mediate activation of all three signaling pathways described above [36], [39], and they play a central role in the activation and proliferation of renal fibroblasts leading to renal fibrogenesis in a variety of pathological settings [40], [41], [42], [43]. We examined the effect of suramin on the activation of these two receptors. PDGFR receptor and its active form (p-PDGFR) were not detectable in the normal kidney; however, renal ablation induced their expression (Fig. 8). In contrast, a small amount of total EGFR and its active form (p-EGFR) was detectable in the normal kidney, and its expression was increased in the remnant kidney (Fig. 8). Although administration of suramin totally blocked renal ablation-induced phosphorylation of PDGFR and EGFR, expression of total PDGFR and EGFR were not affected by this treatment (Fig. 8). This data supports the role of suramin as a potent inhibitor for multiple growth factor receptors and also suggests the importance of PDGFR and EGFR in mediating the anti-fibrotic action of suramin.

### Suramin inhibits Smad-3 phosphorylation in the remnant kidney

**Figure 9:**
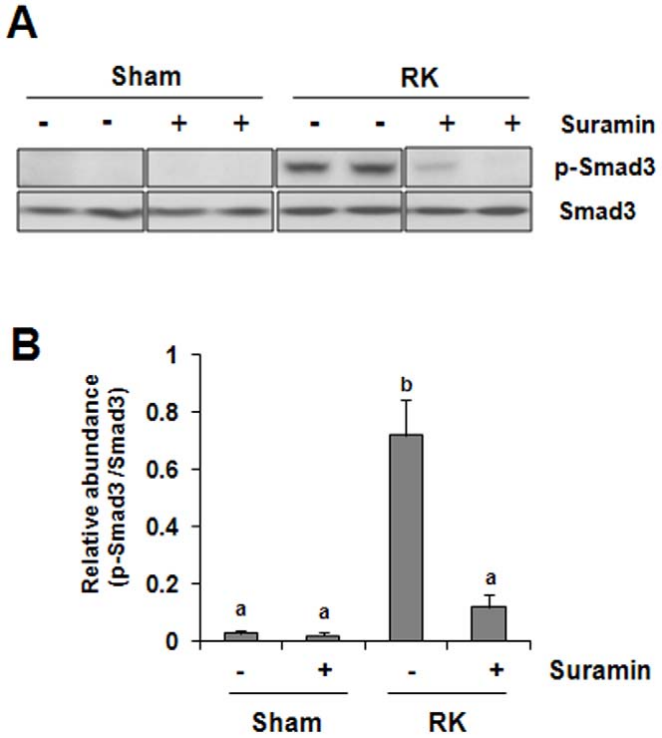
Effect of suramin on phosphorylation of Smad-3 phosphorylation in rat remnant kidney. Kidney tissue lysates were subjected to immunoblot analysis with specific antibodies against phospho-Smad (p-Smad), and Smad (A). Expression levels of p-Smad was quantified by densitometry and normalized with Smad(B). Data are represented as the mean ± S.E.M (n = 6). Means with different superscript letters are significantly different from one another (*P*<0.05).

TGF-betasignaling is associated with almost all forms of kidney disease characterized by renal fibrosis and inflammation, and smad-3 is a critical component of the intracellular pathway that transmits TGF-beta signals from the cell surface into the nucleus [44], [45]. To determine whether suramin would have an inhibitory effect on the TGF-beta signaling pathway, we examined the effect of suramin on Smad-3 phosphorylation in the remnant kidney. Fig. 9 shows that renal ablation induced Smad-3 phosphorylation, which was suppressed by suramin administration. Phosphorylated smad-3 was not detectable in the sham–operated kidney with/without administration of suramin. Total Smad-3 expression was the same in each group. Thus, suramin substantially blocked TGF-beta signaling, which may mediate the antifibrotic effects of this compound in the remnant kidney.

## Discussion

Renal fibrosis is the final common pathway for most forms of progressive renal disease and involves glomerular and vascular sclerosis, and/or interstitial fibrosis. Most renal disorders (whether glomerular or tubulointerstitial, congenital or acquired) lead to renal fibrosis. Thus, there is a great interest in identifying and developing drugs to prevent or reverse fibrosis. Recently, we demonstrated that suramin inhibits activation and proliferation of renal interstitial fibroblasts and attenuates the development of renal interstitial fibrosis when given either immediately after injury or 3 days after initiation of injury in UUO model [12], [13], suggesting it may be a potential drug for treatment of fibrotic disease. However, it remains unclear whether suramin also improves glomerular and vascular lesions as these pathological changes are not typical in UUO model. As the remnant kidney is characterized by the slow development of glomerulosclerosis, vascular sclerosis, tubulointerstitial fibrosis and renal inflammation [14], [15], mimicking human disease, we further tested the efficacy of suramin in this model. Our results revealed that suramin administration is effective in preventing the development of glomerular and vascular sclerosis and attenuating inflammatory response.

To our knowledge, we are the first to demonstrate that suramin exhibits a renoprotective effect in a model of hypertenisve glomerulosclerosis. However, the mechanims by which suramin protects against development of glomerulosclerosis are not fully understood. Although blood pressure is an important factor associated with this process [46], we recently observed renoprotection with suramin in the remnant kidney that was independent of effects on systemic hypertension [12]. This indicates that suramin may attenuate sclerosis through nonhemodynamic factors. Among numerous nonhemodynamic factors, macrophages have been shown to play an important pathogenic role in the progression of glomerulosclerosis. For example, glomerular infiltration of macrophages was observed within 1 to 2 wk of renal ablation, before the development of renal scarring [47]. Glomerular macrophage depletion by X-irradiation has been shown to ameliorate the progression of the glomerular injury in the remnant kidney [15]. Further, macrophage-conditioned medium has been reported to promote prosclerotic responses in cultured rat mesangial cells [48]. In the current study, we examined the effect of suramin on the macrophage infiltration in the kidney and found that suramin treatment significantly suppresed macrophage infiltration as revealed by both histochemistry and immunoblot analysis. This suggests that a reduction of macrophage by suramin may thus have contributed to amelioration of sclerosis.

Suramin-mediated suppression of macrophage infiltration may be through inhibition of chemokine expression. Previous studies have indicated that production of MCP-1 and RANTES is able to build up the chemokine gradient that serves as a chemotactic signal to attract macrophages to the injured site in the kidney [49]. In the present study, we observed increased expression of MCP-1 and RANTES in the remnant kidney, and suramin treatment significantly reduced their expression. Furthermore, suramin inhibited renal ablation-induced phosphorylation of NF-κB, a key transcriptional factor involved in the expression of multiple proinflammatory chemokines and cytokines, including MCP-1 and RANTES. In addition, STAT3, another trancriptional factor, is also highly phosphorylated in the remnant kidney, and its phosphorylation is inhibited by suramin. This suggests that the production of cytokines and chemokines in the damaged kidneys can be transcriptionally regulated by both NF-kappaB and STAT3. In support of this hypothesis, we have recently demonstrated that the inhibition of STAT3 by S3I-201 suppressed the expression of TNF-alpha, IL-1beta, and ICAM-1 without affecting that of MCP-1 in the mouse kidney injured by ureteral obstruction. Since NF-kappaB is well known to play an important role in regulating MCP-1 expression [50], these findings suggest that NF-kappaB and STAT3 can operate in concert to regulate expression of cytokines and chemokines in injured kidneys.

It should be emphasized that NF-kappaB and STAT3 may regulate expression not only of cytokines/chemokines, leading to inflammatory responses, but also other genes involving renal fibrogenesis. Recently, we reported that STAT3 activity is required for the expression of TGF-beta1 and TGF-beta1 receptor II [27]. NF-kappaB has also been shown to be able to interact with AP-1 to regulate the expression of collagen, fibronectin and TGF-beta1, enhancing ECM accumulation [51]. Notably, AP-1 transcriptional factor is subjected to regulation by the ERK1/2 signaling pathway, and suramin treatment also blocked ERK1/2 phosphorylation in the remnant kidney. Therefore, it is likely that suramin inhibits the inflammatory response in the fibrotic kidney through multiple signaling pathways and transcriptional factors.

It is well known that suramin exhibits its pharmacological effects by interfering with the interaction of multiple growth factors/cytokines and their receptors [5], [6], [7], [8].. Previous studies have also shown that suramin is a potent antagonist of PDGF, EGF and TGF-beta receptors whereas activation of these three receptors is strongly associated with differentiation of renal fibroblasts to myofibroblasts, resulting in the production of excessive amount of ECM proteins [52]. Thus, interference with these receptors by suramin and subsequent blockade of signaling pathways initiated from these receptors may be an important mechanism in inhibiting renal fibroblast activation and attenuating production of ECM proteins. Our studies in this model clearly demonstrated that suramin treatment can abrogate phosphorylation of EGF and PDGF receptors and reduce phosphorylation of Smad-3, a key intracellular signaling molecule, downstream of TGF-betareceptors, supporting its inhibitory effect on multiple profibrotic receptors. These results are consistent with our observation in the UUO model [12], providing a strong rationale to develop suramin as a therapeutic intervention for fibrotic kidney disease.

Suramin has been used to treat selected malignancies and metastatic diseases Our previous and current studies reveal that suramin administration can attenuate interstitial fibrosis, reduce glomerular and vascular injury, improve renal function and reduce proteinuria [12]. Further, delayed administration of suramin was also effective in attenuating the progression of renal fibrosis in obstructive nephropathy [13]. Therefore, there is the potential clinical application of suramin as an anti-fibrotic treatment in patients with chronic kidney disease. As suramin is already approved for use in humans, it would be possible to use it in clinical studies in CKD to test its efficacy and safety in patients with chronic kidney injury.

In summary, we have shown that suramin inhibits glomerular and vascular sclerosis. The beneficial effects of suramin are linked to suppression of macrophage infiltration, inhibition of chemokine expression and inactivation of transcriptional factors. As renal fibrogenesis is associated with the increased production of numerous cytokines/growth factors and subsequent activation of their receptors and signaling pathways, suramin with its ability to inhibit the interaction of multiple cytokine/growth factor with their receptors may offer improved therapeutic benefits in kidney fibrotic diseases.

## Materials and Methods

### Chemicals and Antibodies

Antibodies to p-STAT3, STAT3, p-ERK1/2, ERK1/2, p-EGFR, p-PDGFR-beta, PDGFR-beta, p-p65NF-kappaB, p65NF-kappaB were purchased from Cell Signaling Technology (Danvers, MA). Antibodies to GAPDH, EGFR, MCP-1, and RANTES were purchased from Santa Cruz Biotechnology, Inc. (Santa Cruz, CA). ED-1 antibody was purchased from Serotec (Oxford, UK). Suramin and all other chemicals were from Sigma (St. Louis, MO).

### Remnant Kidney Models and Suramin Treatment

The remnant kidney model was created in male Sprague-Dawley rats that weighed 180 to 200 g (Charles River Laboratories, Wilmington, MA). Five-sixths of normal renal mass was surgically ablated according to our previous protocols [12]. Briefly, 20 animals underwent subtotal nephrectomy involving right subcapsular nephrectomy and infarction of approximately two-thirds of the left kidney by ligation of the posterior and one or two anterior extra renal branches of the renal artery. In addition, 12 rats underwent a sham operation (laparotomy and manipulation of the renal pedicles). On the second week after surgery, rats were randomly divided into suramin treatment and non-treatment groups. Suramin was given at 10 mg/kg once per week for two weeks. 24-hour urine samples were collected in metabolic cages starting at day 1 and weekly for the determination of urinary levels of protein. 28 days after surgery, all animals were sacrificed and the kidneys were then collected for further analysis.

### Immunoblot Analysis

Immunoblot analysis of tissue samples was conducted as described previously [53]. The densitometry analysis of immunoblot results was conducted by using NIH Image software (National Institutes of Health, Bethesda, MD).

### Immunohistochemical Staining

Immunohistochemical staining was performed according to the procedure described in our previous studies [53]. ED1, MCP-1, and RANTES staining were semi quantified by a computer-aided morphometric analysis (MetaMorph; Universal Imaging Co., Downingtown, PA). Briefly, a grid containing 117 (13X9) sampling points was superimposed on images of cortical high-power field (×400). The number of grid points overlying positive area (except tubular lumen and glomeruli) were counted and expressed as a percentage of all sampling points, as described previously [27]. For each kidney, 10 randomly selected, non-overlapping fields were analyzed in a blinded manner.

### Statistical Analysis

All the experiments were conducted at least three times. Data depicted in graphs represent the means ± SEM for each group. Inter-group comparisons were made using one-way analysis of variance (ANOVA). Multiple means were compared using Tukey's test. The differences between two groups were determined by Student t-test. Statistical significant difference between mean values was marked in each graph. P<0.05 is considered significant.
